# Ferulic Acid From Plant Biomass: A Phytochemical With Promising Antiviral Properties

**DOI:** 10.3389/fnut.2021.777576

**Published:** 2022-02-07

**Authors:** Io Antonopoulou, Eleftheria Sapountzaki, Ulrika Rova, Paul Christakopoulos

**Affiliations:** Biochemical Process Engineering, Division of Chemical Engineering, Department of Civil, Environmental and Natural Resources Engineering, Luleå University of Technology, Luleå, Sweden

**Keywords:** antiviral activity, nutraceuticals, plant biomass, extraction, ferulic acid, metabolism, enzymatic derivatization, immunity

## Abstract

Plant biomass is a magnificent renewable resource for phytochemicals that carry bioactive properties. Ferulic acid (FA) is a hydroxycinnamic acid that is found widespread in plant cell walls, mainly esterified to polysaccharides. It is well known of its strong antioxidant activity, together with numerous properties, such as antimicrobial, anti-inflammatory and neuroprotective effects. This review article provides insights into the potential for valorization of FA as a potent antiviral agent. Its pharmacokinetic properties (absorption, metabolism, distribution and excretion) and the proposed mechanisms that are purported to provide antiviral activity are presented. Novel strategies on extraction and derivatization routes, for enhancing even further the antiviral activity of FA and potentially favor its metabolism, distribution and residence time in the human body, are discussed. These routes may lead to novel high-added value biorefinery pathways to utilize plant biomass toward the production of nutraceuticals as functional foods with attractive bioactive properties, such as enhancing immunity toward viral infections.

## Introduction

The emerged global COVID-19 outbreak has led to significant efforts by the international research community for the development of novel vaccines, drugs and treatments to fight the pandemic. In parallel with vaccine development, already approved drugs and several natural bioactive compounds have been screened for their inhibitory activity against viruses in order to repurpose them for treatment (1, 2). It has been quite evident that better protection procedures, with high immune efficacy against viral infection, could play a significant role in future societal responses and preparedness to fight epidemics. In particular, the use of plant-derived nutraceuticals, not necessarily as drugs, but as food supplements with protective, curative and inactivating effects toward a variety of viral infections, may contribute to combat future pandemics or diseases that can affect not only a patient's heath but also their quality of life.

Ferulic acid (FA, 4-hydroxy-3-methoxycinnamic acid) is a promising phytochemical with strong antioxidant activity, whose antiviral potential has been underlined during the past years. It has a broad spectrum of other attractive biological properties including UV absorptive, skin whitening, antibacterial, anti-inflammatory, anti-thrombosis, neuroprotective and antitumor effects that highlights its importance for human health, nutrition and well-being (3, 4). Studies suggest that phenolics, such as FA, may have a vital role for combating human, animal or plant viruses.

Additional to its low cytotoxicity and appreciable bioavailability score, FA is also a CYP1A2 inhibitor, which increases its drug half-life and averts serious drug interactions (5). CYP1A2 is a member of the cytochrome P450 mixed-function oxidase system and is involved in the metabolism of xenobiotics in the body involved in drug metabolism. Thus, FA could be a great candidate with good pharmacokinetic properties for control of viral infections.

In this review article, we will focus on the abundance, pharmacokinetics and proposed antiviral mechanisms of FA, while the potential of reported plant extracts containing FA as major metabolite and synthesized FA derivatives will be explored for their effect against a wide variety of human, animal and plant viruses. These valorization routes may lead to novel high-added value pathways to utilize plant biomass and respective residues toward the production of nutraceuticals with attractive bioactive properties.

## Occurrence of FA in Plant Biomass

**Figure 1:**
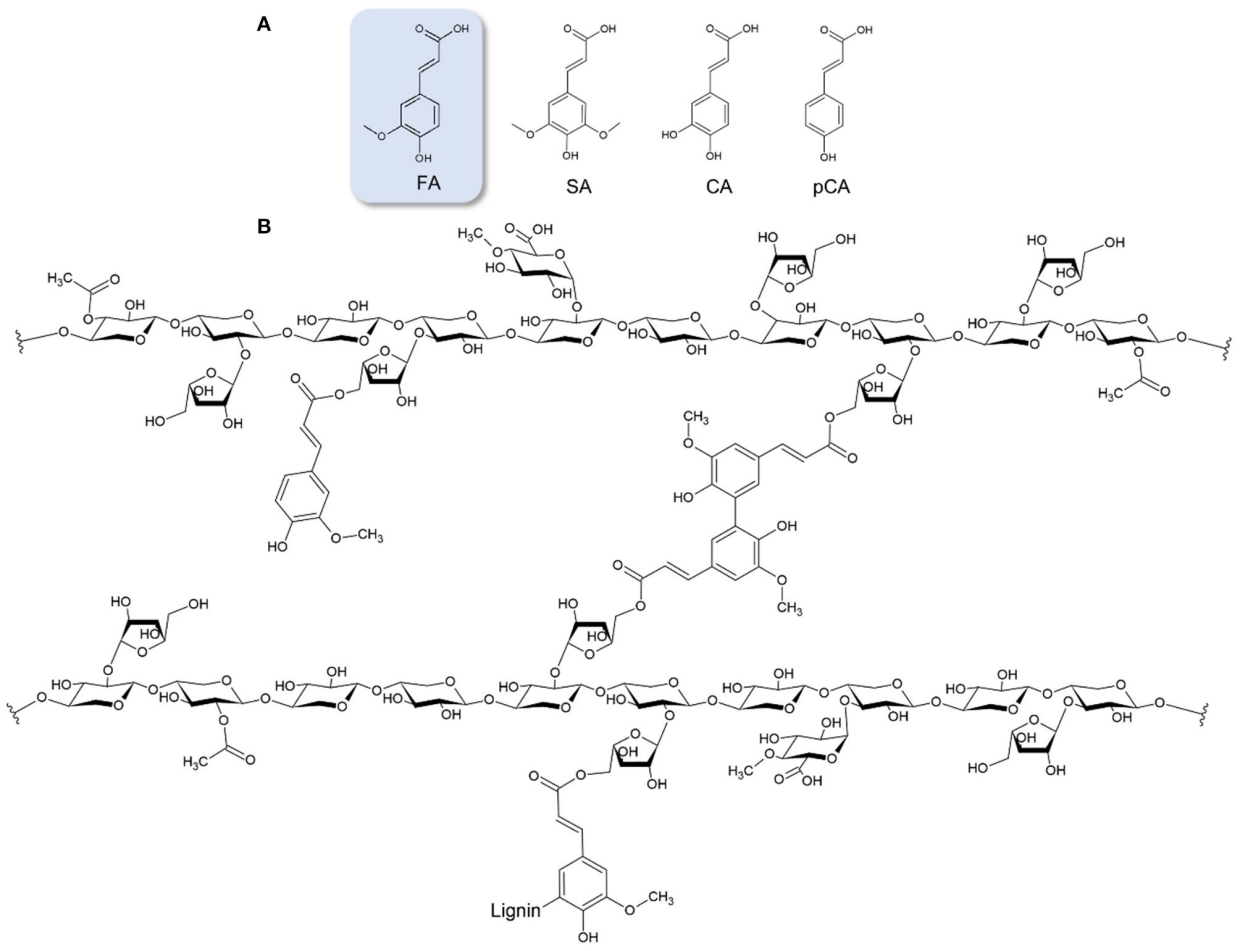
**(A)** Hydroxycinnamic acids; FA, ferulic acid; SA, sinapic acid; CA, caffeic acid; pCA, *p*-Coumaric acid. **(B)** Example of feruloylated glucuronoarabinoxylan, where FA is esterified to the *O*-5 hydroxyl group of a-L-arabinofuranose. Cross-linkage between hemicellulose chains is an example of a 5,5′ FA dehydrodimer. FA linkage with lignin is also demonstrated.

FA is the most abundant hydroxycinnamic acid found in plant biomass, followed to a lesser extent by *p*-coumaric acid (Figure 1A). It can be found in plant cells walls, mainly in its t*rans*-form, usually esterified or etherified to the polymers within the lignocellulosic matrix. FA is commonly found in commelinid plants (rice, wheat, oats and pineapple), grasses, grains, vegetables, flowers, fruits, leaves, beans, seeds of coffee, artichoke, peanut and nuts (6). In graminaceous monocots, such as wheat and rice, FA can be found up to a 3% w/w concentration and esterified to the *O*-5 hydroxyl group of FA and α-L-arabinofuranose in glucuronoarabinoxylan (7) (Figure 1B). In few dicots, such as spinach or sugar beet, it is found at a concentration up to 1% w/w and esterified in pectin to the *O*-2 or *O*-5 hydroxyl group of α-L-arabinofuranose in arabinan or to the *O*-6 hydroxyl group of β-D-galactopyranose in (arabino-)galactan, both of which are side chains in rhamnogalacturonan I (8). FA has been found also esterified to the *O*-4 group of α-D-xylopyranose in xyloglucans of bamboo (9). FA can be oxidatively cross-linked to form intermolecular ester bonds to another arabinoxylan and ether-ether bonds between hemicellulose and lignin. Different diferulic acids are detected; 8,5′-, 5,5′-, 8,4′-, 8,8′- and less commonly 8,5′-(benzofuran)- and 8,8′-(aryl)-diFA (10), while FA trimers and tetramers have been also identified (11). Cross-linking of cell wall polysaccharides and lignin by hydroxycinnamic acids leads to a dramatic increase in mechanical strength, decelerates wall extension, and acts as barrier to block infection. FA is also detected in all families of gymnosperms, being ester-linked to the primary cell walls at a concentration between 0.01 and 0.16% w/w (12). In cereals, FA also exists in ester forms such as steryl ferulates (γ-oryzanol), which was firstly recognized in 1954 in rice bran oil. Several methods have been recognized to extract FA by hydrolysis of γ-oryzanol (13).

## Extraction and Purification of FA From Plant Biomass

To utilize FA for its bioactive properties, recovery in a form of extract or in a purified form is necessary (14). Extraction and further purification of FA and other *p*-hydroxycinnamic acids found in plant biomass can be done by different methods. Purification strategies can lead to obtaining the bioactive compound in pure form for direct use as bioactive agent or as a donor for further derivatization to synthesize novel agents with improved properties.

If FA is free esterified and not bound on the cell wall matrix, a direct extraction step can be applied in order to recover the valuable compound. Efficient extraction with green solvents has been reported, such aqueous ethanol solution (15) or hot water (16). Application of physical activation, such as ultrasound or microwave, may enhance extraction yields. If FA is encased in the cell wall matrix, pretreatment methods are usually applied, such as alkaline hydrolysis or enzymatic hydrolysis. Alkaline hydrolysis is a traditional procedure that is less green as it employs harsh chemicals and high temperatures, thus may have limitations for use in the cosmetic, pharmaceutical and food industries. Enzymatic hydrolysis of esterified FA can be done by the use of feruloyl esterases (EC 3.1.1.73), which consist accessory lignocellulose degrading enzymes (17–20). This class of enzymes, in nature, catalyzes the hydrolysis of the ester bond between FA and α-L-arabinofuranose in hemicellulose, releasing FA and degrading the crosslinking between hemicellulose and lignin. As lignocellulose is highly recalcitrant and in order to improve accessibility of feruloyl esterases to release *p*-hydroxycinnamic acids, use of a consortium of cellulases and hemicellulases in often required. Use of enzymes has many advantages, such as high specificity leading to fewer degradation products, recyclability and preservation of the substrate, while the price of lignocellulose-degrading enzymatic cocktails has dropped significantly during the years making the process more affordable (21).

Hydrolysis methods often result in complex liquors due to the release of sugars, proteins, and other phenolic compounds. Thus, purification steps are needed to further increase extract purity. Purification of FA has been reported by ethanol washing (22, 23), adsorption on adsorbents, such as activated charcoal (22, 24), resins (25–30) and magnetic nanoparticles (31), membrane filtration (32–36) and chromatographic methods, such counter-current high performance liquid chromatography (CCC) (37, 38). Examples on the aforementioned techniques are presented in Table 1.

**Table 1:** Examples of reported methods on the pretreatment of plant biomass and further purification of FA.

**Plant source**	**Pretreatment step**	**Purification step**	**Yield/Purity/Efficiency**	**References**
Wheat bran	Defatted with hexane, alkaline hydrolysis, acidification to pH 2, extraction with ethyl ester (three times)	Counter-current chromatography followed by pH-zone-refining counter-current chromatography	98–99% purity	(38)
Brewer's spent grain	Dilute sulfuric acid hydrolysis followed by alkaline hydrolysis	Ethanol extraction	46.17 mg/100 g BSG	(23)
Sugar cane bagasse	Alkaline hydrolysis	Adsorption by powdered activated charcoal and further treatment by anion macroporous resin exchange chromatography	FA could be totally washed out by 0.2 mol/L NaOH, high purity obtained	(24)
*Radix Angelicae Sinensis*	Microwave-assisted ethanol extraction	High-speed counter-current chromatography	>98% purity	(37)
Wheat bran	Enzymatic hydrolysis	Nanofiltration membranes (MWCO between 600 and 800 g mol^−1^)	85% retention for FA	(34)
Destarched wheat bran	Alkaline hydrolysis	Adsorption on Amberlite XAD4 resin	49.9 mg FA/g resin, 99.5% recovery using methanol as eluent	(27)
Wheat bran	Ultrasound-assisted ethanol extraction	Adsorption on magnetic nanoparticles	77.9–97.5% recovery	(31)
Corn fiber	Alkaline hydrolysis	Ultrafiltration membrane (Nadir UP 150)	98.0% rejection of arabinoxylans	(35)
Corn bran	Alkaline hydrolysis	Adsorption on mesoporous carbon	Much higher adsorption capacity for FA than those of activated charcoal, other activated carbon materials, and macroporous resins	(39)
Sugar beet pulp, maize bran, wheat bran, rice bran	Enzymatic hydrolysis	Adsorption on Amberlite XAD-4 resin	1.9/5.5 mg FA/g	(29)
Brewer's spent grain	Alkaline hydrolysis	Adsorption by synthetic resin (Lewatit VPOC1064 MD PH®)	Partial purification	(28)
Wheat bran	Enzymatic hydrolysis	Demineralization by homopolar electrodialysis, adsorption on weak anion-exchange resin (Amberlyst A21- Dow)	52% of FA was released by enzymatic hydrolysis, of which 83% was crystallized with purity 90–95%	(26)
Flax shives, wheat bran and corn	Alkaline hydrolysis, pressurized	Ultrafiltration and ethanol solubilization/centrifugation cycles (2 times)	56% efficiency, clean TLC and FTIR (FA)	(32)
Corn bran	Alkaline hydrolysis	Ultrafiltration (5,000 Da MWCO), nanofiltration	91.8% recovery, 8.47 g FA crystals/kg corn bran with 84.45% purity	(36)
Grass	Dilute sulfuric acid followed by alkaline hydrolysis	Ethanol extraction	30.9 g FA/100 g grass	(22)
		Adsorption with activated charcoal or polyvinylpolypyrrolidone (PVPP)	3.3 g FA/100 g grass	
			0.5 g FA/100 g grass	
Sugar beet pulp	Twin-screw extraction and enzymatic hydrolysis	Microfiltration (3 μm), adsorption on activated charcoal	50% purity (FA), 6 cycles to achieve 97% efficiency	(33)
Wheat bran	Enzymatic hydrolysis	Adsorption on weak anionic resin	15 g/L, 67% recovery	(25)

## Pharmacokinetics of FA: Absorption, Metabolism, Distribution and Excretion

**Figure 2:**
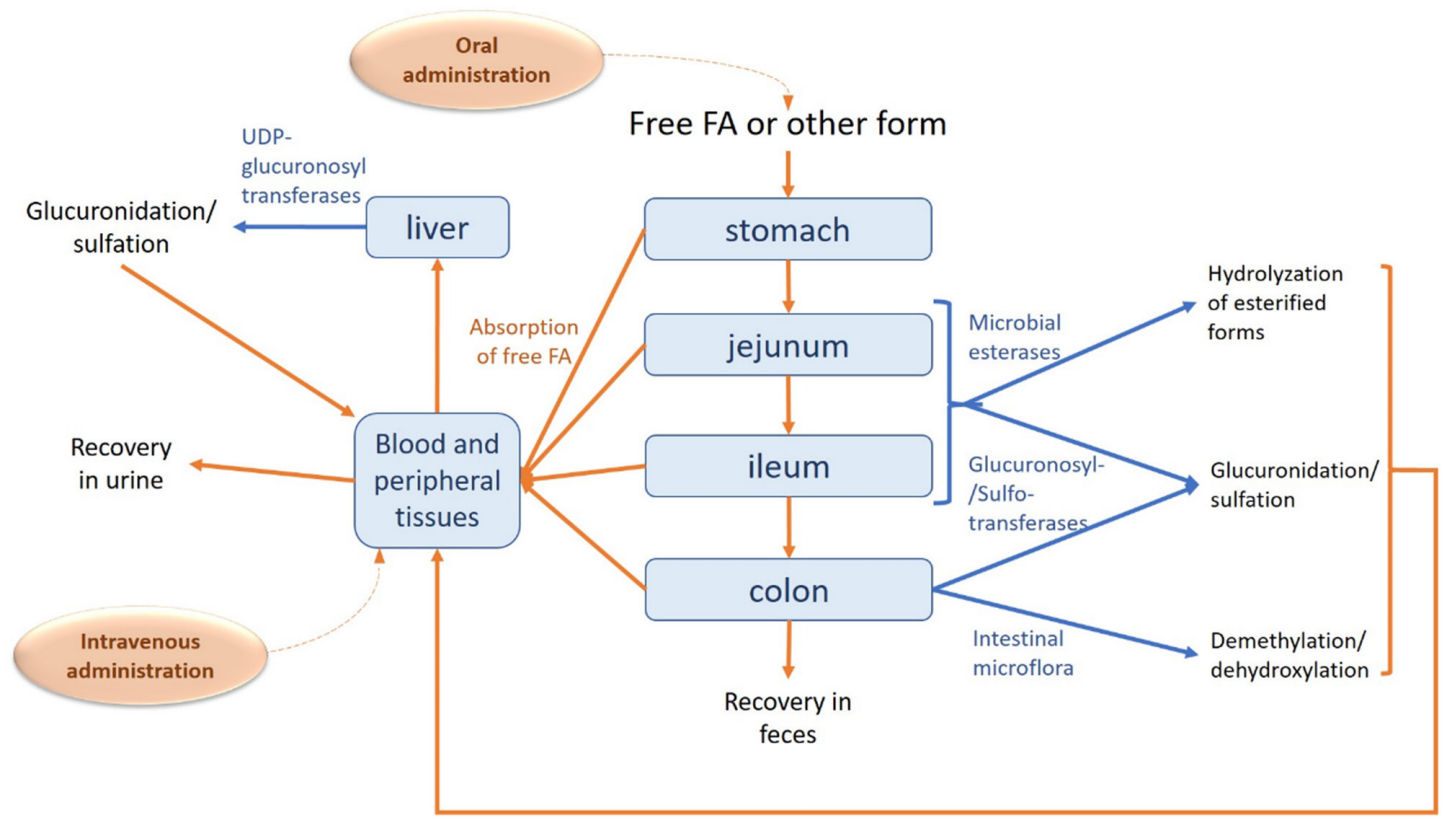
Absorption, metabolism and excretion of FA in human body. The route of FA is described with orange arrows, while the metabolic modifications are shown in blue.

When ingested, FA can be absorbed in the stomach (40), small intestine; jejunum (41, 42) and ileum (42), and colon (43) (Figure 2). However, the major and most important site for FA absorption in the human body is the colon, where microbial esterases in the lumen of the intestine hydrolyze the esterified FA from the food matrix to generate free FA. Administration of FA sugar esters, such as 5-*O*-feruloyl-L-arabinofuranose or feruloyl-arabinoxylan, leads to the same metabolites being present in plasma as when free FA is administrated, reinforcing the belief that FA esters are broken down to FA and then follow the same metabolic route (44). However, there is also a hypothesis supporting that FA sugar esters are absorbed intact and then hydrolyzed in mucosal cells or in circulation. In any case, research shows that free FA can be absorbed before reaching the ileum, but when bound to fiber, it has to be degraded in the hindgut in order to be absorbed and further metabolized (45).

Studies on an *in vitro* model for human colonic epithelium showed that FA is mainly transported in its free form, after released from its ester form by esterases. FA is suggested to be absorbed by a combination of mechanisms: passive transcellular diffusion and facilitated transport. Transcellular diffusion is dependent on the pH of the environment, the pKa and the hydrophobicity of the compound. FA, with a pKa equal to 4, could be transported in an undissociated form *via* a passive diffusion mechanism in the low gastric pH environment (46). However, this might not be the case for intestinal absorption, as FA would not maintain its undissociated form in the weak acidic environment of the intestine (pH > 5) (47).

Studies regarding FA absorption in rats through the jejunal mucosa have indicated a potential mechanism involving a Na(+)- dependent transport mechanism (41), whereas Itagaki et al. (48) also mention the possibility of a H+-dependent carrier, testing the uptake of FA in Caco-2 cells. However, such transporters have not been identified yet. According to Konishi and Shimizu (49), transepithelial transport of FA across Caco-2 cell monolayers was suggested to be related to binding to a monocarboxylic acid transporter (MCT), in particular to MCT1. However, in one of their more recent studies it is eventually supported that FA transport is achieved through another MCT type transporter and not MCT1 (50). MCTs are believed to be involved in gastric absorption, apart from the colonic, but the specific type of transporter has not been determined (51). Both of the previously mentioned mechanisms give free FA on the serosal side, with only a small number of conjugated forms.

FA is further metabolized mainly by the liver, where the major metabolites are FA-conjugated forms. The major metabolite of FA is FA-sulfoglucuronide, with other common conjugated forms being FA-glucuronide and FA-sulfate (45). These kind of conjugations is a way of detoxifying the compound and increasing its polarity (52). Additional FA metabolites include *m*-hydrophenylpropionic acid, feruloylglycine, dihydroferulic acid, vanillic acid, vaniloylglycine, feruloyl-sulfate and trans-feruloyl-4-*O*-β-D-glucuronide (43, 53). The conjugation reaction for the formation of FA-sulfoglucuronide, FA-glucuronide and FA-sulfate is carried out by the activities of sulfotransferases and UDP glucuronosyl transferases, while the intestinal mucosa and kidney may take part in this process. Conjugation of FA may be dose dependent as very high doses of FA may saturate the conjugation enzymes, leading to accumulation of free FA in the plasma. Apart from conjugation, metabolic reactions include reduction of the double bond, demethylation, dehydroxylation of C3 or C4 (54, 55), as well as methylation (53, 56). The efflux of the conjugates is also transporter(s)-dependent (57).

Maximum plasma concentration as investigated in humans is reached 24 min after ingestion (57), whereas Li et al. (58) report a much lower value (2 min) in rats. Half-lives for the distribution and elimination phases in the latter case have been calculated at ~10 and 60–106 min, respectively. It is suggested that the amount of FA that does not proceed to the liver, ~50% of the initial intake, is found in the bloodstream, gastric mucosa and peripheral tissue (59). Another study reports that from an initial intake of FA (521 μmol/kg), 14 μg/g was detected in the heart, 82 μg/g in the kidney, 22 μg/g in the spleen, 34 μg/g in the lung, 28 μg/g in the liver, 15 μg/g in the uterus and 2.6 μg/g in the brain of rats, 30 min post-administration (60). The main distributor of FA in the body seems to be serum albumin (61). The source, form, and amount of FA ingested influences the ratio of the different forms available in blood for further uptake by the tissues. According to a different study, total plasma FA concentration in rats included no more than 24 % free FA, and 76% of glucuronidated and sulfoglucuronidated forms which peaked 30 min after oral administration (52).

In humans, excretion of FA via urine reaches a plateau after ~7–9 h after ingestion (62). The recovery of free FA in urine was reported to be around 5% in both rats and humans (63), whereas the excretion of total FA, both in its free form as well as in its conjugated forms, mostly as feruloyl glucuronide, reaches 11–25% (62). The percentage of the conjugated, and more specifically sulfoglucuronidated FA, compared to the total excreted amount is even higher in urine than in plasma, reaching 84% (52). Only 0.5–0.8% of ingested FA has been found in feces of rats, indicating very efficient absorption rate for FA (64, 65).

Zhao et al. (40) suggest that oral administration of free FA results in a quick absorption, but also quick excretion of FA, whereas administration through a capsule would contribute to maintaining the desirable concentration of FA in the body for a longer period of time. For instance, a four times enhancement of the bioavailability of encapsulated FA compared to free form has been observed in rats (66). Ou and Kwok (67) report that the most effective way for FA to enter enterohepatic circulation, as tested in rats, is to be ingested in its natural form, as part of plant tissue. However, Adam et al. (65) mention that the recovery of FA is low when it has been consumed in the form of whole cereal, indicating that absorption is higher when FA is in its free form. The bioavailability of FA is affected by the complexity of the polysaccharide matrix to which FA is bound. For example, the presence of diferulic acids is concomitant to cross-links between polysaccharide chains that impede hydrolyzation by the responsible enzymes (68). On the contrary, Rondini et al. (69) observed that urinary excretion of FA after bran consumption was 15-fold less than that after intake of free FA. Such differences of urinary excretion of FA were still observed with a perfusion of rat intestine with pure FA, allowing the recovery of 50% of the intake dose and only 3.2% after bran consumption. Moreover, wheat arabinoxylan, that is rich in esterified FA, has been shown to act as a prebiotic, stimulating growth of good bacteria such as lactobacilli and bifidobacteria in the human gastrointestinal tract (70, 71). Studies on metabolism, distribution and excretion of free FA or derivatives after intravenous administration have not been yet reported.

## Proposed Mechanisms for Antiviral Activity

During the past years, FA has been suggested to play an important protective role against viruses. There are different types of mechanisms responsible for this effect. FA is known to promote induction of heme oxygenase-1 (HO-1) and hence may show utility for boosting type 1 interferon response (IFNs) that regulates the activity of the immune system (72, 73). Sodium ferulate was found to activate toll-like receptors (TRL) 7 and 9 in influenza A-infected mice, which are proteins that recognize viral nucleic acids and rapidly trigger different signaling cascades that contribute to the production of IFNs to antiviral defense. This effect could be secondary to HO-1 induction, and possibly reflects an additional beneficial effect of FA (74).

The anti-inflammatory impact of nutraceuticals, such as FA, might also be expected to quell the excessive inflammatory reaction within lung parenchyma evoked by viral infections, whose lethality is mediated by an acute respiratory distress syndrome. In particular, FA has been reported to hinder the production of inflammatory proteins induced by viral infections, such as macrophage inflammatory protein-2 (MIP-2) in respiratory syncytial virus (RSV)-infected cells (75) and IL-8 in murine macrophage cells infected by influenza (76). Thus, FA could be a natural solution for decreasing such response both by suppressing viral spread, and by dampening pro-inflammatory signaling in endothelial cells that in turn promotes influx of inflammatory cells. Mccarty and Dinicolantonio (77) suggested that a very low daily dosage ranging from 500 to 1,000 mg of FA could aid the control of influenza and corona viruses.

The most important and major impact of FA is its potential to have an inhibiting effect against key viral proteins that are associated with viral functions, such as replication. Two analogs of FA have been proposed to have inhibiting effects to neuraminidase, a surface glycoprotein vital for the viral replication and an important target for anti-influenza drug, using molecular docking (78). Virtual screening of 300 immunomodulatory medicinal compounds revealed that FA, together with only five other natural phenolic compounds (arzanol, genistein, resveratrol, rosmanol and thymohydroquinone), had significant interaction with the SARS CoV-2 viral proteins (SARS coronavirus peptidase (2GTB), severe acute respiratory syndrome coronavirus 2 (SARS CoV-2) protease 3CL (Mpro) (6LU7), spike glycoprotein (6VSB), NSP-9 replicase protein (6W4B) and NSP-15 endoribonuclease (6VWW) by forming hydrogen bonds with the active site residues with low binding energy, inhibiting their activity (79). A study screening 548 anti-viral natural and synthetic antiviral compounds *in silico* also showed that FA together with caffeic acid were found to inhibit a 3D model of SARS CoV-2 membrane protein (80). These studies underline the potential important function of FA as a natural inhibitor of SARS-CoV-2.

Moreover, FA has been found to inhibit viral replication by hindering porcine parvovirus (PPV)-induced intrinsic PK-15 cell apoptosis by suppressing the expression of proteins that play a role in the cell apoptosis pathway (Cyt-c, Apaf-1, Caspase-9) and Bcl-2 group proteins in particular. In addition, FA prevents generation of reactive oxygen species (ROS) by blocking caspase activity (81).

## Antiviral Activity of Plant Extracts Containing FA

Plant extracts have been long known in folk medicine for their therapeutic properties. During the past decades, a variety of extracts rich in bioactive phenolic compounds, where FA is found as main or secondary component, have been evaluated for their activity against viruses. Except for their antiviral effect, these extracts demonstrate a rich profile of attractive bioactive properties, including antioxidant, anti-inflammatory and antiproliferative activities, and could be a valuable asset for boosting our immunity against various conditions, including viral infection. Plant extracts containing FA and evaluated for their antiviral activity come from angiosperms, in particular herbaceous plants with no woody stem above ground, and various flowering plants including shrubs and trees. An overview of the assessed plant extracts is presented in Table 2.

**Table 2:** Antiviral effect of plant extracts containing FA.

**Plant source**	**Extraction method^a^**		**Major detected compounds**	**Virus**	**Antiviral effect**	**Cytotoxicity**	**References**
*Melissa officinalis* L. (lemon balm)	Aqueous extraction of leaves, filtration and spray drying		FA, caffeic acid, rosmarinic acid	HSV-1	Complete viral inhibition after 6 h at maximum tolerable concentration of the extract	Maximum tolerable concentration: 0.25%	(82)
*Cimicifuga heracleifolia* (bugbane)	–		FA, isoferulic acid (pure compounds)	RSV	42.8% lower MIP-2 levels compared to the control at 500 μM FA	Cells >95% viable when treated with 500 μM compound	(75)
*Plantago major* L. (broadleaf plantain)	Successive hot water extraction of whole plant, filtration, concentration in vacuo and lyophilization		Baicalein, baicalin, luteolin, aucubin, caffeic acid, chlorogenic acid, FA, *p*-coumaric acid, oleanolic acid, ursolic acid, vanillic acid (based on literature)	HSV-2	EC_50_ = 843 μg/ml	CC_50_ = 1,809 μg/ml	(83)
	-		FA (pure compound)	ADV-8	EC_50_ = 52.5 μg/ml	CC_50_ = 92.6 μg/ml	
				ADV-11	EC_50_ = 23.3. μg/ml		
*Ficus carica* (fig)	Successive methanol extraction of the plant latex, filtration and evaporation, followed by silica gel chromatography eluting with hexane and after through hexane–ethyl acetate, drying under reduced pressure		Caffeic acid, 3,4-dihydrobenzoic acid, *p*-OH-phenylacetic acid, *p*-coumaric acid, luteolin, N-argenine, FA (majority in the extract)	HSV-1	100% blocked replication of the virus and virucidal activity when incubated with the extract for 1 h with 78 μg/ml extract	No cytotoxic effect at tested concentrations	(84)
				ECV-11			
				ADV			
*Kalanchoe gracilis*	Cold-pressing of leaves, filtration and freeze drying		7.59% w/w FA, 0.33% w/w, quercetin, 0.16% w/w kaempferol	EV71	IC_50_ = 35.88 μg/ml (plaque reduction assay on RD cells)	No cytotoxicity at a range of 1–500 μg/ml	(85)
					IC_50_ = 40.82 μg/ml (cell-based FRET assay)		
				CVA16	IC_50_ = 42.91 μg/ml (plaque reduction assay on RD cells)		
					IC_50_ = 47.87 μg/ml (cell-based FRET assay)		
*Eugenia singampattiana*	Extraction of dried leaves with methanol or water, centrifugation and evaporation–redissolution with distilled water		4-hydroxybenzoic acid, caffeic acid, rutin, FA, coumaric acid epigallocatechin gallate, quercetin, myricetin, kaempferol	PRRSV	Inhibition observed with 25 μg of methanol extract or 75–100 μg of water extract	–	(86)
Propolis	Propolis Extract ACF® (specific purified ethanol extract)		Aromatic acids incl., E-*p*-coumaric acid (main), benzoic acid, FA (~3%), fatty acids, esters incl. benzyl *p*-coumarate, flavonoids, chalcones, dihydrochalcones, sugars	HSV-1	Inhibition of adsorption in cells at 0.1 mg/ml when incubation was ≥ 60 min	CC_50_ = 0.13 mg/ml	(87)
				HSV-1/HSV-2	Virucidal effect for extract concentration ≥ 10 mg/ml and incubation for at least 15 min		
	Ethanol extract, chloroform soluble fraction		Kaempferol, galangin, quercetin, fisetin, chrysin, luteolin, acacetin, caffeic acid, FA, *p*-coumaric acid (based on literature)	HRV-4	IC_50_ = 5.0 μg/ml (SRB method)	CC_50_ = 82.3 μg/ml	(88)
	-		FA (pure compound)	HRV-2	IC_50_ = 175.1 μM	CC_50_ > 5,000 μM	
				HRV-3	IC_50_ = 248.7 μM		
				HRV-4	IC_50_ = 232.3 μM		
*Strychnos minor*	Successive extraction of dried plant with methanol, sonication, vacuum evaporation	Leaves	Gallic acid, FA, o-coumaric acid, quercetin	H1N1	IC_50_ = 46.69 μg/ml (SRB method)	CC_50_ = 1,026 μg/ml	(89)
		Stem		H1N1	IC_50_ = 22.43 μg/ml	CC_50_ = 100 μg/ml	
*Diotacanthus albiflorus*		Leaves		H1N1	IC_50_ = 60.09 μg/ml	CC_50_ = 100 μg/ml	
		Stem		H1N1	IC_50_ = 33.98 μg/ml	CC_50_ = 50 μg/ml	
*Strychnos nux-vomica*		Leaves		H1N1	IC_50_ = 33.36 μg/ml	CC_50_ = 20 μg/ml	
		Stem		H1N1	IC_50_ = 23.60 μg/ml	CC_50_ = 40 μg/ml	
*Cayratia pedata*		Leaves		H1N1	IC_50_ = 65.99 μg/ml	CC_50_ = 100 μg/ml	
		Stem		H1N1	IC_50_ = 20.50 μg/ml	CC_50_ = 18.30 μg/ml	
*Hibiscus sabdariffa* (roselle)	Calyses extracted in deionized distilled boiling water and filtered		Anthocyanins, phenolic acids, organic acids, saponins, and alkaloids, including malic acid, protocatechuic acid and FA (based on literature)	FCV-F9	Viral titer undetectable after 15 min with 40 or 100 mg/ml	Cytotoxicity observed at > 40 mg/ml	(90)
				MNV-1	Viral titer undetectable after 24 h with 40 mg/ml		
				HAV	Viral titer undetectable after 24 h with 40 mg/ml		
	–		FA (pure compound)	FCV-F9	Viral titer undetectable after 15 min after 3 h with 0.5 or 1 mg/ml	–	
				MNV-1	0.94 logPFU/ml reduction after 24 h at 1 mg/ml		
				HAV	No significant reduction in viral titers after 24 h		
*Eucaluptus camaldulensis* (river red gum)	Extraction of dried stem bark with acetone, filtration and drying by evaporation		Benzoic acid, quinol, myricetin, salicylic acid, rutin, ellagic acid, vanillic acid, *p*-hydroxybenzoic acid, chlorogenic acid, syringic acid, *o*-coumaric acid, FA (4.56 mg/100 g), gallic acid, vanillin, cinnamic acid, *p*-coumaric acid etc.	TMV	72.22% inhibition (protective effect) 50.43% inhibition (curative effect) 33.33% inhibition (inactivating effect) at 100 μg/ml	–	(91)
*Humulus lupulus* (hops)	Hydroalcoholic extract of female inflorescences		Chlorogenic acid, t-FA (0.078 μg/mg), gallic acid, *p*-OH benzoic acid, rutin, syringic acid	Influenza (PR8, pH1N1, NWS, ULSTER)	IC_50_ = 99 μg/ml for the PR8 strain	Cell viability > 90% for 20–140 μg/ml	(92)
*Berberis lyceum* (barberry)	Extraction of leaves with 70% methanol, filtration and drying by vacuum evaporation		Quercetin, myricetin, gallic acid, caffeic acid, FA (0.37 mg/g DE)	HCV	IC_50_ = 11.44 μg/ml	CC_50_ > 200 μg/ml	(93)
*Licopersicum esculentum* (tomato)	Leaf extraction in 95% ethanol: 5% acetic acid, filtration and evaporation		Chlorogenic acid, caffeic acid, FA (0.0195 mg/100 g DW), gallic acid, rutin, quercetin	MS2	3.47 logPFU/ml (reduction of viral titer)	–	(94)
				Av-05	5.78 logPFU/ml		
*Solanum tuberosum* (potato)	Mixing of dried peels with 95% ethanol: 5% acetic acid, filtration, sonication of the residue with ethanol, filtration of the sonicated residue, mixing of the two supernatants and concentration		Gallic acid, chlorogenic acid, caffeic acid, FA (3.29 mg/100 g DW), rutin, quercetin	MS2	3.9 logPFU/ml (reduction of viral titer)	–	(95)
				Av-05	2.8 logPFU/ml		
*Haplophyllum tuberculatum*	Extraction of the whole plant with ethanol (soaking method), filtration and evaporation		Gallic acid, FA (26.86 mg/kg), catechin, quinol, syringic acid, caffeic acid, vanillic acid, ellagic acid, cinnamic acid, *o*-coumaric acid, benzoic acid, *p*-hydroxy benzoic acid	TMV	65.38% inhibition (protective effect); 95.73% inhibition (inactivating effect)	–	(96)
*Garcinia mangostana* (mangosteen)	–		FA isolated from the extract	H1N1	IC_50_ = 140 μg/ml	CC_50_ = 702 μM	(78)
Rice bran	–		Isolated cycloartenol ferulate	HIV-1	IC_50_ = 2.2 μg/ml (reverse transcriptase inhibition assay)	–	(97)
			Isolated 24-methylenecycloartanol ferulate				
					94% inhibition (4 mg/ml)		
	–			HIV-1	IC_50_ = 1.9 μg/ml		
					89% inhibition (4 mg/ml)		
*Gallesia gorazema* (pau-d'alho)	Soaking of dried plant roots with dichloromethane		α-Amyrin, tetradecanal, palmitic acid, octadecanal, γ-sitosterol, β-amyrin, 28-hydroxyoctacosyl ferulate	HSV-1	93% inhibition (protective effect at 100 μg/ml)	CC_50_ >200 μg/ml	(98)
	–		28-hydroxyoctacosyl ferulate isolated from extract	HSV-1	EC_50_ = 21.6 μg/ml	CC_50_ >200 μg/ml	
				HSV-2	EC_50_ = 86.5 μg/ml		
*Vaccinium oldhamii* (wild blueberry)	Extraction of dried fruits with 80% ethanol, concentration and elution with 40% ethanol through column chromatography		Procyanidin B2, unspecified ferulic acid derivatives, two ferulic acid *O*-hexosides, quercetin-3-*O*-rhamnoside and quercetin-*O*-pentoside-*O*-rhamnoside	IFV	IC_50_ = 22 μg/ml (viral inhibition plaque assay)	CC_50_ = 160 μg/ml	(99)

a *Conditions or fractions that offer highest antiviral effect in the respective study; HSV, herpes simplex virus; RSV, respiratory syncytial virus; ADV, adenovirus; ECV, echovirus; EV, enterovirus; CV, coxsackievirus; PPRSV, porcine reproductive and respiratory syndrome virus; HRV, human rhinovirus; H1N1, influenza A virus subtype; FCV, feline calicivirus; MNV, murine noronivirus; HAV, hepatitis A virus; TMV, tobacco mosaic virus; HCV, hepatitis C virus; MS2, Av-05, bacteriophage; HIV, human immunodeficiency virus; IFV, influenza virus; EC_50_, half-maximal effective concentration; the concentration required to obtain a 50% inhibitory effect, IC_50_, half-maximal inhibitory concentration; the concentration at which the number of viral plaques is reduced by 50%, CC_50_, half-maximal cytotoxicity concentration, the concentration that reduced cell viability to 50% of the control; DW, dry weight; DE, dry extract*.

Lemon balm (*Melissa officinalis* L.; Lamiaceae) is a perennial herbaceous plant of the mint family that has been used in traditional medicine for nervous complaints, lower abdominal disorders and for treating Herpes lesions. Among lemon balm leaf extracts containing FA, caffeic acid and rosmarinic acid as key extractives, highest virucidal effect against simple herpex virus type 1 (HSV-1) was observed for the aqueous extract, where the infection activity was repressed completely at 6 h after treatment at maximum tolerable concentration in infected rabbit kidney (RK) cells (82). More recently, the inhibitory effect of lemon balm hydroalcoholic extract has been demonstrated for HSV type 2 (HSV-2) replication by the cytopathic effect inhibition assay on Vero cells (100) and for adenovirus (ADV) evaluated on HEp2 cells using the MTT (3-[4,5-dimethylthiazol−2-yl]-2,5-diphenyltetrazolium bromide) assay (101).

FA, as a main active constituent of *Cimicifuga heracleifolia* extract, along with isoferulic acid, has also been reported as active against respiratory syncytial virus (RSV) by reducing the virus-induced production of inflammatory proteins MIP-2. The levels of MIP-2 observed on infected murine macrophage cells were reduced to 74.4 and 42.8% of the control at pure FA concentrations of 50 and 500 μg/ml respectively, whereas isoferulic acid reduced levels to 58.0 and 35.6% of the control at the respective concentrations (75). *Cimicifuga* rhizoma methanol extract has also been found to suppress viral replication of mouse hepatitis virus (MHV-A59), with a half maximal effective concentration (EC_50_) of 19.4 μg/ml calculated by the plaque assay (102). The rhizoma of *Cimicifuga* species (bugbane; Ranunculaceae), including *C. heracleifolia* and *C. dahurica* has been used traditionally as an antipyretic, analgesic, anti-inflammatory agent in East Asian countries (103).

The herbaceous perennial broadleaf plantain (*Plantago major* L.; Plantaginaceae) is popular in traditional Chinese medicine and has long been used for treating various diseases varying from cold to viral hepatitis. The aqueous extract of the whole plant, along with pure compounds representing the five main classes of biologically active compounds found in its extracts (benzoic acids, flavonoids, iridoid glycoside, hydroxycinnamic acids and triterpenes), have been investigated for their ability to suppress herpesviruses and adenoviruses (ADV). Interestingly, the aqueous extract of the whole plant showed only inhibitory activity against HSV-2 while pure FA was promising against ADV-8 and ADV-11, showing an EC_50_ = 52.5 and 23.3 μg/ml respectively, evaluated with the XTT assay on basal cell carcinoma cells. Among hydroxycinnamic acids, caffeic acid was the most active, achieving inhibition for HSV-1, HSV-2 and ADV-3 but not ADV-8 and ADV-11, followed by chlorogenic acid, which showed activity against all the mentioned viruses (83).

FA was also highlighted to be the major constituent in hexanic and hexane-ethyl acetate extracts of fig latex (*Ficus carica*; Moraceae). The two extracts proved to inhibit viral entry into Vero cells, since they were incubated with them prior to inoculation with HSV-1, echovirus type 11 (ECV-11) and ADV. Inhibition of the virus was successful also when the extracts were incubated with infected cells, as well as when they were incubated with the virus and then brought in contact with the cells. Thus, *F. carica* extracts could block viral DNA (for HSV-1 and ADV) and RNA (for ECV-11) replication and also exhibit virucidal activity (84).

*Kalanchoe* (Crassulaceae) are succulent plants mainly native to Madagascar and tropical Africa. In traditional medicine, *Kalanchoe* species have been used to treat infections, rheumatism and inflammation. Leaf extract from *Kalanchoe gracilis*, containing 7.59 % w/w FA, exhibited antiviral activity against RNA enteroviruses enterovirus 71 (EV71) and coxsackievirus A16 (CVA16) that was demonstrated by an half maximal inhibitory concentration (IC_50_) of 35.88 and 42.91 μg/ml, respectively, using the plaque reduction assay on RD cells. Further *in vitro* investigation revealed that the leaf extract achieved inhibition of viral protease 2A that is present in both viruses, showing an IC_50_ value of 40.82 μg/ml for EV71 and 47.87 μg/ml for CVA16 employing the cell-based FRET assay (85). Another plant with antiviral properties in whose extract FA has been detected as a secondary metabolite, is *Eugenia singampattiana*, a critically endangered small tree belonging to the Myrtaceae family that is endemic in India. Extracts of the leaves were used to treat Marc-145 cells infected with porcine reproductive and respiratory syndrome virus (PRRSV). The methanol and water extracts achieved suppression of the virus at a concentration of 25 and 75-100 μg, respectively (86).

Two bacteriophages used as surrogates for human enteric viruses, MS2 and Av-05, were inhibited by tomato extracts (leaf, stems, roots, whole plant) containing FA, although gallic acid was the major identified phenolic acid. Virucidal assay using 5 mg/ml of the leaf extracts resulted in 3.47 and 5.78 logPFU/ml reduction of MS2 and Av-05 titers respectively for Pitenza cultivar, and 3.78 and 4.93 logPFU/ml reduction of the viral titers for Floradade cultivar (94). Potato peel (*Solanum tuberosum*) acidified ethanol extract, containing 3.29 mg FA/100 g dry weight, was also tested with a standardized plaque assay against the two bacteriophages and reduced viral titer by 3.9 logPFU/ml for MS2 and 2.8 logPFU/ml for Av-05 when incubated with the viruses for 3 h with 5 mg/ml (95).

The aqueous extract of the red calyces of *Hibiscus sabdariffa* (roselle; Malvaceae) has achieved undetectable viral titers at a concentration of 40 mg/ml, when tested against feline calicivirus (FCV-F9), murine norovirus (MNV-1) and hepatitis A virus (HAV). FA was also tested separately, together with protocatechuic acid and malic acid as main components of the extract. FA at a concentration of 1 mg/ml exhibited promising results, inhibiting two viruses to the point where they could not be detected after 3 h (FCV-F9) and 24 h (MNV-1). However, FA did not have an inhibitory effect against HAV (90). Methanolic leaf extract of the medicinal plant *Berberis lyceum* (barberry; Berberidaceae) containing FA, successfully inhibited replication of hepatitis C virus (HCV), with IC_50_ = 11.44 μg/ml calculated in infected HepG2 cells. The extract also seems to significantly inhibit the expression of the viral non-structural protein NS5A by 42 and 61% at 100 and 200 μg/ml (93).

*Eucaluptus camaldulensis* bark extract, containing 4.56 mg/100 g FA among other phenolic acids and flavonoids, was able to protect, cure from and inactivate tobacco mosaic virus (TMV), as determined utilizing the half leaf method on *Nicotiana glutinosa* leaves. When used to treat the leaves at a concentration of 100 μg/ml 24 h before infection, the extract offered 72.2% inhibition of the virus. Although the most promising results yielded for the protective effect of the extract, it also had curative and inactivating antiviral action, with the respective inhibition percentages reaching 50.43 and 33.33% (91). In another study by the same research group, ethanol extract of the whole plant from *Haplophyllum tuberculatum* (Rutaceae) had both protective and inactivating effect for TMV (96). The extract contained FA among other phenolic acids.

FA is also one of the major phenolic compounds in the hydroalcoholic extract of *Humulus lupulus* (hops) that was found active against various strains of the influenza virus (PR8, pH1N1, NWS, ULSTER). Antiviral assays performed on Madin-Darby Canine Kidney (MDCK) and A549 lung carcinoma cells showed considerable potential of the plant extract, as it inhibits replication of the PR8 strain with an IC_50_ of 99 μg/ml. Anti-influenza effect was tested when the extract was added to the culture in different stages of infection (before, during and after). Viral replication was partly inhibited when virus was incubated with extract before infection, suggesting a direct effect on the virus. By adding extract to the cells during the infection, a significant reduction of the viral titer was noticed for PR8, NWS and ULSTER strains (46, 50 and 29% respectively). Addition after infection resulted in 75, 44 and 29% inhibition, respectively. The highest inhibitory potential, against all the viral strains examined, was calculated in the cases where the extract was present in all the phases of the infection (92). It was speculated that since the extract was able to restore the reducing conditions of infected cells, by increasing glutathione content, its antiviral activity might be also due to an interference with redox-sensitive pathways required for viral replication.

*In vitro* studies in MDCK cells, testing the antiviral activity of methanol extracts of medicinal plants from South India against influenza A virus subtype H1N1, indicate promising properties of leaf and stem extracts from *Strychnos minor, Diotacanthus albiflorus, Strychnos nux-vomica* and *Cayratia pedata*. The extracts mainly comprised of gallic acid, FA, *o*-coumaric acid and quercetin. The metabolite content was predominantly different among different samples, while although total phenolic content was related to positive influence on viral inhibition, a negative correlation of FA concentration was considered in respect with antiviral activity (89). Hariono et al. (78) also report inhibitory activity of FA isolated from *Garcinia mangostana* (mangosteen; Clusiaceae) against the neuraminidase of H1N1 (IC_50_ = 140 μM). Mangosteen has been known in traditional medicine mostly in Southeast Asia.

Although not a plant extract, propolis, that incorporates exudates gathered from tree buds, sap flows or other botanical sources, has been shown to contain FA as an important constituent. A patented ethanol extract from Canadian propolis, that was harvested from beehives located in an area rich in poplar trees, has been shown to be effective toward HSV-1 and HSV-2 when tested for its prevention of viral adsorption in MDBK cells and virucidal effect (87). The anti-rhinovirus activity of Brazilian propolis and its constituents has also been evaluated. Among various fractions of propolis ethanol extract, the replication of human rhinovirus type 4 (HRV-4) in HeLa cells was limited using a chloroform- and ethyl acetate-soluble fraction by IC_50_ = 5.0 and 5.2 μg/ml respectively (88). In the same study, pure FA inhibited three HRV types with an IC_50_ ranging between 175.1 and 248.7 μM, showing better performance than the tested drug reference ribarivin. Phenolic acids, such as caffeic and *p*-coumaric acid, as well as flavonols and flavonones had also superior inhibitory effect.

FA derivatives with antiviral activity has been isolated from plant sources. For example, cycloartenol ferulate and 24-methylenecycloartanol ferulate are two compounds found in rice bran that effectively inhibit human immunodeficiency virus 1 (HIV-1) reverse transcriptase, with a considerable IC_50_ value of 2.2 μM and 1.9 μM, respectively (97). Moreover, De Jesus Silva Júnior et al. (98) have isolated an FA derivative identified as 28-hydroxyoctacosyl ferulate from the dichloromethane root extract of *Gallesia gorazema* (Petiveriaceae), a shrub widely distributed in the Atlantic Rain Forest of Brazil. The ester was tested against HSV-1 and HSV-2 viruses, inhibiting both by 99.8 and 97.8% and with a half-maximal cytotoxicity concentration equal to 21.6 and 86.5 μg/ml, respectively, whereas the crude root extract inhibited only HSV-1 by 93%. The ester shows a much higher selectivity for HSV-1 than HSV-2, which could be a strong indication that it is responsible for the antiviral activity of the extract against HSV-1. Unspecified FA derivatives are pointed out as major contributors to the anti-influenza activity of an extract from Japanese wild blueberries (*Vaccinium oldhamii;* Ericaceae), preventing the adsorption or cell entry of the virus in MDCK cell cultures. Dry fruit extracts purified through column chromatography with different percentages of ethanol were screened for their antiviral activity and the lowest adsorption inhibitory concentration was calculated to have an IC_50_ value of 22 μg/ml (99).

## FA Derivatives as Antiviral Agents

### Chemically Synthesized Derivatives

FA derivatives could enhance the bioactive property of the molecule and potentially influence the distribution and time of stay in the human body. The first reported attempt on chemical derivatization of FA targeting enhancement of its antiviral activity was based on a polymerization strategy to create dehydrogenated polymers that resemble natural structures like lignin. Different size synthesized polymers of FA and *p*-coumaric acid showed antiviral activity against HIV-1 through two mechanisms; by inhibiting cytopathicity induced by the virus and blocking its protease, as examined in MT-4 cells. The four FA polymer fractions, with a size range of 500 Da to over 30 kDa, exhibited HIV-1 protease inhibition with IC_50_ values between 3.2 and 0.9-1.0 μg/ml. It was noticed that the larger the polymer, the lower the IC_50_ value of the compound (104).

Later, several strategies on FA amidation were explored. Spasova et al. (105) recruited peptide chemistry using coupling reagent EDC/HOBt to create amides of 3-aminomethyl glaucine linked to HCAs, including FA. The derivatives were screened for their activity against enteroviruses Poliovirus type 1 (LSc-2ab), CV-B1 and EV-13 and HRV-14. Overall, all 3-aminomethylglaucine derivatives of FA, sinapic, *o*- and *p*- coumaric acids proved to have antiviral properties against HRV-14 and were not cytotoxic at respective IC_50_ concentrations, worthing further investigation.

Huang et al. (106) synthesized a series of substituted FA amide and the corresponding hydrogenated derivatives targeting increased antiviral effect against plant viruses and in particular TMV. Among compounds from both series, the ones that preserved the double bond in the carbon chain had higher activity overall. The best generated compound had protective and curative activity of 40.7 and 46.4 % respectively, in infected *N. tabacum* leaves. Cui et al. (107) also tested very similar hydrogenated FA derivatives that were synthesized chemically via hydrogenation of FA using Pd/C as catalyst, to produce hydrogenated ethyl ferulate as intermediate, and subsequent substitution with 2-amino-1-phenylethanol derivatives. The two most active compounds had *p*-benzyloxy substitution in the R^1^ position and propynyl or allyl substitution in the R^2^ position respectively. They resulted in a protective effect against TMV of 15.2 and 22.8%, respectively and a respective curative effect of 28.9 and 20.7%.

Activity against the two plant viruses, TMV and cucumber mosaic virus (CMV) has been detected in α,β-unsaturated amide derivatives of FA with an α-aminophosphonate moiety. EC_50_ was observed to be 216.30 and 284.6 μg/ml for TMV and CMV respectively, for a compound with R^1^ = 4-Cl and R^2^ = 4-CF_3_-Ph, which was superior to that of ningnanmycin. All 26 synthesized compounds showed antiviral activity to some level, which was observed to be reinforced by the presence of an electron- withdrawing group at the 2- or 4- position of the aromatic ring (108). TMV has also been the target for synthesis of 23 conjugates containing an acylhydrazone moiety that were tested *in vivo* against the virus and a concentration of 500 μg/ml. The majority of them exhibited better inactivating activity than the commercial drug ribavirin. Protective activity ranged from 14.5 to 58.7%, curative activity from 17.4 to 54.2% and inactivating activity from 59.3 to 94.2%. The compound with the most prominent properties was the one with R^1^ = PhCH_2_ and R^2^ = 2-Th substitutions, as it had 18.8% protective, 23.0% curative, 94.2% inactivating activity and EC_50_ equal to 36.59 μg/ml regarding its inactivating activity; almost three times lower than ribavirin (109).

FA sulfonamides are very potent against tobacco mosaic virus (TMV). Antiviral assay conducted with the half-leaf method resulted in curative activity ranging between 30.2–57.9 %, protection activity between 31.2–59.7 % and inactivation activity 43.7–87.3 % for the different FA derivatives at a concentration of 500 μg/ml. The most prominent compound inactivated TMV with an EC_50_ of 84.8 μg/ml, significantly lower than the one of widely used drug ribavirin. Results showed that, as far as structure is concerned, electropositive substituents of the 4-position of the sulfonamide benzene ring are more active than electronegative when it comes to curating effect, whereas the opposite is observed in terms of protection from the virus (110).

Tang et al. (111) have chemically synthesized myricetin derivatives with an FA amide substitution and investigated their activity against TMV. The curative and protective effect of the conjugates at 500 μg/ml was measured using the half-leaf method on *N. tabacum L*. leaves. Results varied, from 15.8 to 55.5% for the curative effect and 5.3 to 62.1% for the protective effect of the different derivatives. Further testing for the most promising compounds using preliminary bioassays resulted in calculations of EC_50_ values of 196.11, 425.3 and 386.7 μg/ml for the protection activity of the best compounds, with the R substitutions being 3,4-di-OCH_3_, 3,4-di-Cl and 4-Br, respectively (*n* = 3). The compound with the 4-Br substitution also had an EC_50_= 472.4 μg/ml regarding its curative activity. All the aforementioned results were comparable to commercial antiviral agent ningnanmycin. FA amino derivatives have been tested against their inhibitory activity of H1N1 neuraminidase, showing encouraging results. More specifically, pure FA has an inhibitory effect described by IC_50_ = 140 μM, while when nitrate substitutes C3, IC_50_ is reduced to 127 μM. The best results (IC_50_ = 90 μM) were provided by an ethyl FA ester, with an isopropyl substitution at C2 and a nitrate at C3 (78).

FA esters with a quinazoline group are also effective against TMV and CMV when tested *in vivo* on *N. tabacum* leaves and *Chenopodium amaranticolor* leaves, respectively. Overall, the most effective compound was the one with the following substitutions: R^1^= 2-OCH_3_-Ph and R^2^= 4-oxoquinazolin-3(4H)-yl-methyl. It showed 60.8, 78.2 and 90.8% curative, protective and inactivating activity against TMV and 58.1, 69.8 and 78.2% for CMV respectively (112). Synthesis and screening of 35 designed trans-FA esters with a chalcone group for their anti-TMV activity was performed by Gan et al. (113). The results were encouraging, as 5 compounds showed better curative and inactivating effect and 7 compounds had higher protective activity than commercially used antivirals, when tested at a concentration of 500 μg/ml with an antiviral biological assay on *N. tabacum*. FA is also reported as a precursor for the chemical synthesis of derivatives containing a dithioacetal moiety. A series of such compounds has been tested for their activity against TMV, and the most successful hit achieved higher antiviral activity than commercial virucidal ribavirin (62.5% curative, 61.8% protective and 83.5% inactivating activity at a concentration of 500 μg/ml) (114).

### Potential for Enzymatic Derivatization

So far, the vast majority of FA conjugation methods has been based on chemical synthesis routes. However, a major drawback of these methods is the requirement for toxic or expensive catalysts, strong acids, high temperatures and long reaction times, resulting in low yields that may not be possible to compensate for the related energy cost (115). In addition, the heat sensitivity and oxidation susceptibility of FA makes such processes more difficult to control (116). Enzymatic derivatization is a developing field of research that introduces a more sustainable solution, applying milder conditions and resulting in exceptional selectivity, due to the use of enzymes as biocatalysts.

A demonstrated route for enzymatic FA derivatization, targeting enhancement of antiviral activity, is via (trans)glycosylation. The only current example is the use of rutinase, an enzyme that naturally catalyzes the hydrolyzation of rutin to quercetin and rutinose, but it can be used for the reverse reaction, aiming to the formation of glycoside derivatives. Katayama et al. (117) have studied the enzymatic synthesis of phenolic acid rutinosides aided by a rutinase derived from tartary buckwheat seeds. Although among vanillic, sinapic, FA and caffeic acid rutinosides, sinapic acid rutinoside had the highest antiviral activity. FA rutinoside also exhibited antiviral activity against FCV in an antiviral assay conducted with the MTT method, preventing the reduction of cell viability after infection. In all cases, conjugation of the phenolic acids with rutin enhanced their anti-FCV activity. This reaction has demonstrated that, since use of FA is often limited by weak solubility in either hydrophilic or hydrophobic formulations, glycosylation with a more hydrophilic compound can be a potent strategy to alter its solubility and cell penetration ability, as well as its biological activity.

A potential route for the enzymatic derivatization of FA resulting in enhancement of antiviral properties is via (trans)esterification. Triaglycerol lipases (EC 3.1.1.3) are most commonly used due to their broad specificity toward lipids and extraordinary robustness in low water media, the latter being required for the formation of esters over hydrolysis (118). There are various reports on the synthesis of FA-based esters employing lipases, including the acylation of FA with ethanol (119), trinolein or trilinolenin (120), glycerol (121, 122), arbutin (123), vitamin E (124), phytosterols (125), quercetin (126) and β-sitosterol (127). In particular, β-sitosteryl ferulate is mentioned as a potential inhibitor of SARS-CoV-2 main protease 3CLpro, where molecular docking simulations showed a promising binding mode and a binding energy of−7.8 kcal/mol for the ester-protease complexes (128). Feruloyl esterases (EC 3.1.1.73) consist another interesting esterase subclass that, in nature, catalyze the hydrolysis of the ester bond between α-Larabinofuranose and FA or other hydroxycinnamic acids, present in lignocellulose (129, 130). Although they are not so stable in low-water content media as lipases (131), their ability as biocatalytic tools for (trans)esterification has been demonstrated. Their major advantage over lipases is the specificity toward hydroxycinnamic acids, allowing acylation of FA with a wide range of hydrophilic or lipophilic substitutions, such as alcohols, polyols or sugars (132–137).

Last, a promising route is via oxidation/crosslinking employing laccases (EC 1.10.3.2), which oxidize phenols to produce phenoxy radicals that are then polymerized. The oligomers or polymers that occur as products have been reported to have enhanced antioxidant properties (138) and can be a promising alternative to chemical polymerization (104).

Although products from the aforementioned proposed routes have not been evaluated yet *in vitro* for their antiviral potential, such synthesis routes may result in a highly diverse portfolio of FA based antiviral agents, offering beneficial effects toward boosting our immunity against viral infections. Overall, there are a lot of possibilities for enzymatic modification of FA that not only may enhance its antiviral potential, but also potentially enable a different fate in the metabolic route, having an impact on its absorption site and retention time in the human body. A summary of the synthesized FA derivatives assessed for their antiviral potential is presented in Table 3.

**Table 3:** Antiviral effect of FA derivatives.

**Derivative**	**General structure**	**Virus**	**Antiviral activity/Cytotoxicity of the compound**	**Structure**	**References**
**Chemically synthesized**					
FA polymers	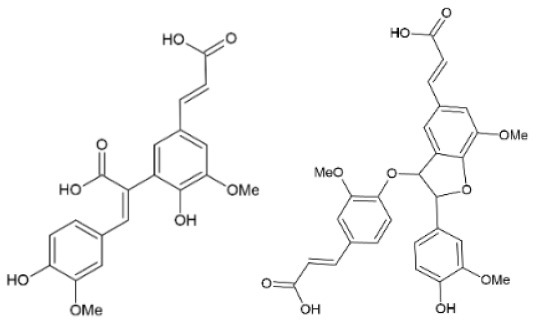	HIV-1	IC_50_ = 1.0 μg/ml IC_50_ = 0.9 μg/ml IC_50_ = 2.7 μg/ml IC_50_ = 3.2 μg/ml	>30 kDa 30–10 kDa 10–1 kDa 1 kDa−500 Da	(104)
	Example: diferulate, triferulate				
FA amide of 3-aminomethyl glaucine	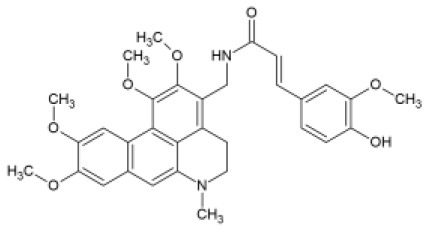	HRV-14	IC_50_ = 12.00 μM CC_50_= 113 μM	-	(105)
FA amides	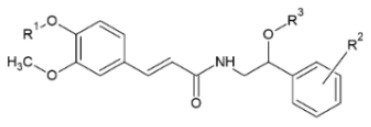	TMV	40.7% protective effect 46.4% curative effect	R^1^: n-Pr; R^2^: H R^3^: propynyl	(106)
Hydrogenated FA amides	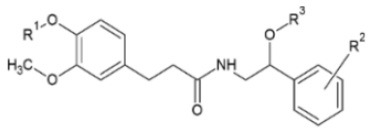	TMV	27.3% protective effect 30.4% curative effect	R^1^: Me R^2^: *p*-Cl R^3^: propynyl	(106)
	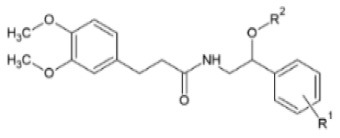	TMV	15.2% protective effect 28.9% curative effect	R^1^: *p*-benzyloxy R^2^: propynyl	(107)
α,β-unsaturated amide derivatives of FA with an α-aminophosphonate moiety	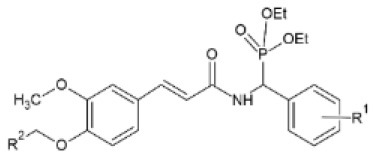	TMV	EC_50_ = 180.37 μg/ml (protective effect)	R^1^: 4-Cl	(108)
			EC_50_ = 285.42 μg/ml (curative effect)	R^2^: 4-CF_3_-Ph	
		CMV	EC_50_ = 216.30 μg/ml (protective effect)		
			EC_50_ = 284.67 μg/ml (curative effect)		
Trans-FA derivatives containing acylhydrazone moiety	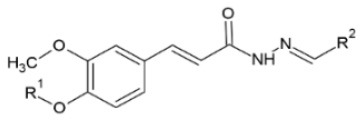	TMV	18.8% curative effect	R^1^: PhCH_2_	(109)
			23.0% protective effect	R^2^: 2-Th	
			94.2% inactivating effect		
			EC_50_ = 36.59 μg/ml		
FA sulfonamides	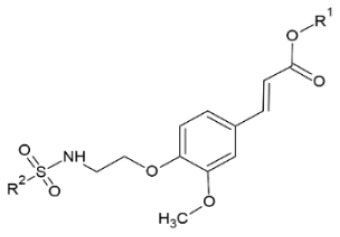	TMV	39.8% curative effect	R^1^: -C_2_H_5_	(110)
			59.7% protective effect 87.3% inactivating effect	R^2^: 4-NO_2_-Ph	
			EC_50_ = 84.80 μg/ml		
Myricetin derivatives with an FA amide scaffold	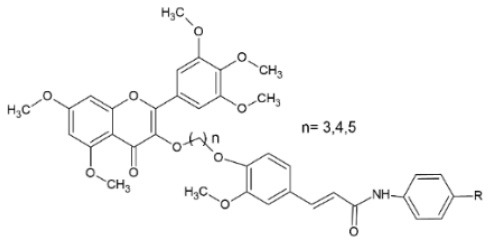	TMV	55.5% curative effect 53.3% protective effect EC_50_ = 472.4 μg/ml	R: 4-Br, *n* = 3	(111)
FA 3-amino derivatives/esters	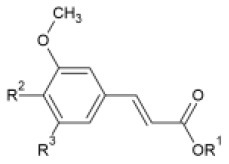	H1N1	IC_50_ =90.0 μg/ml	R^1^: CH_2_(CH_3_)	(78)
				R^2^: CH(CH_3_)_2_	
				R^3^: NO_2_	
FA derivatives with a quinazoline moiety	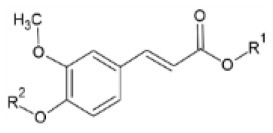	TMV	60.8% curative effect	R^1^: 2-OCH_3_-Ph	(112)
			78.2% protective effect	R^2^: 4-oxoquinazolin-3(4H)-yl-methyl	
			90.8% inactivating effect		
		CMV	58.1% curative effect	R^1^: 2-OCH_3_-4-allyl-Ph	
			69.8% protective effect		
				R^2^:4-oxoquinazolin-3(4H)-yl-methyl	
			78.2% inactivating effect		
Trans-FA esters with a chalcone group	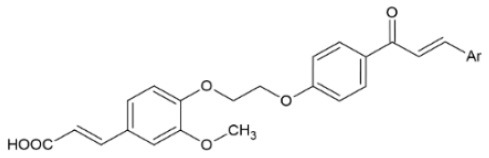	TMV	63.9% curative effect	R: Me	(113)
			64.6% protective effect	Ar: 2-F-Ph	
			92.3% inactivating effect		
			EC_50_ = 214.20 μg/ml		
FA derivatives containing dithioacetal moiety	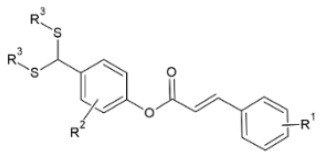	TMV	62.7% curative effect	R^1^: 3-OCH_3_-4-OCOCH_3_	(114)
			52.3% protective effect		
			73.8% inactivating effect	R^2^: 2-OCH_3_	
			EC_50_=73.7 μg/ml	R^3^: -(CH_2_)_2_OH	
	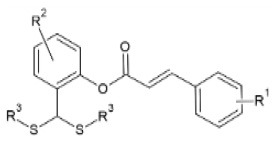	TMV	62.5% curative effect	R^1^: 3-OCH_3_-4-OCOCH_3_	
			61.8% protective effect	R^2^: H	
				R^3^: -(CH_2_)_2_OH	
			83.5% inactivating effect		
			EC_50_= 50.7 μg/ml		
					
	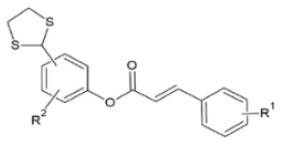	TMV	48.% curative effect	R^1^: H	
			48.% protective effect	R^2^:H	
			53.% inactivating effect		
			EC_50_=355.6 μg/ml		
**Enzymatically synthesized**					
FA rutinoside	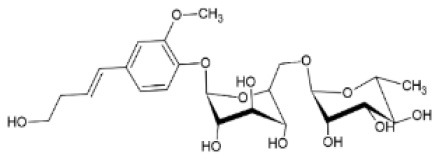	FCV	40% increase of cell viability	–	(117)
FA β-sitosterol ester	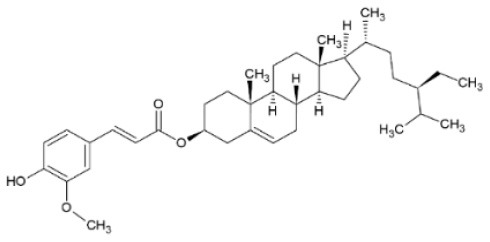	SARS-CoV-2	Binding energy = −7.8 kcal/mol (molecular docking simulation with SARS-CoV-2 3CLpro)	–	(127)
					(128)

*HIV, human immunodeficiency virus; HRV, human rhinovirus; TMV, tobacco mosaic virus; CMV, cucumber mosaic virus; H1N1, influenza A virus subtype; FCV, feline calicivirus; SARS-CoV-2, severe acute respiratory syndrome coronavirus; EC_50_, half-maximal effective concentration; the concentration required to obtain a 50% inhibitory effect; IC_50_, half-maximal inhibitory concentration; the concentration at which the number of viral plaques is reduced by 50%; CC_50_, half-maximal cytotoxicity concentration, the concentration that reduced cell viability to 50% of the control*.

## Conclusions

**Figure 3:**
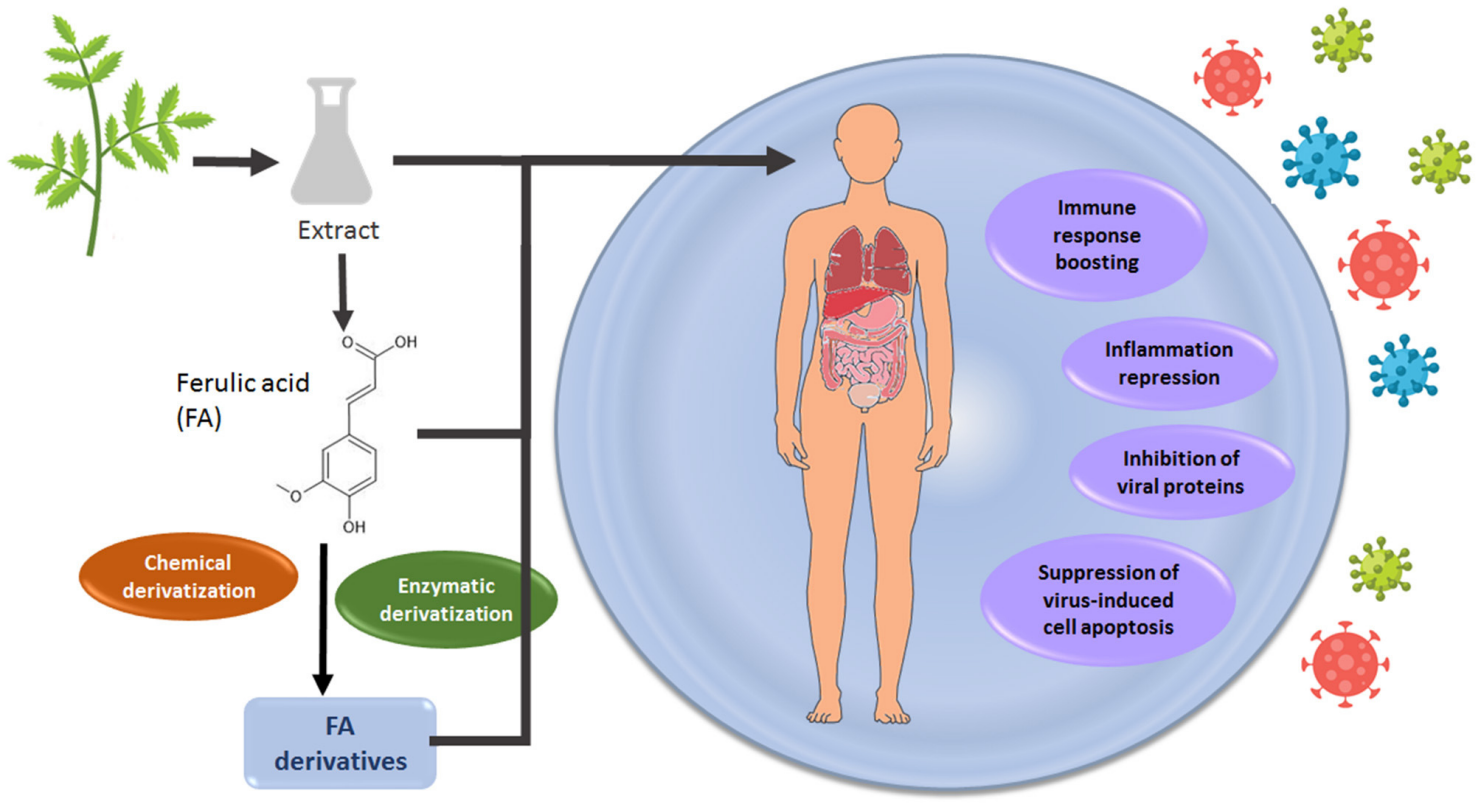
A route for FA valorization toward developing antiviral agents.

Extraction of FA from plant biomass and its residues and further derivatization for nutraceutical applications could be a viable and high-added value pathway within biorefineries. More systematic studies are expected as a continuation of *in vitro* studies, in order to confirm the therapeutic effect and distribution of FA derivatives to protect target cells against viruses. Nevertheless, the use of nutraceuticals, such as FA, may aid toward boosting immunity and aid protection from viral infections, which can be a valuable asset for future epidemics and new viruses. An overview of the potential route for FA valorization is presented in Figure 3.

## Author Contributions

IA and ES conceptualized and wrote the review paper. UR and PC conceptualized the review paper and provided feedback on the manuscript. All authors contributed to the article and approved the submitted version.

## Conflict of Interest

The authors declare that the research was conducted in the absence of any commercial or financial relationships that could be construed as a potential conflict of interest.

## Publisher's Note

All claims expressed in this article are solely those of the authors and do not necessarily represent those of their affiliated organizations, or those of the publisher, the editors and the reviewers. Any product that may be evaluated in this article, or claim that may be made by its manufacturer, is not guaranteed or endorsed by the publisher.
